# A178 ELUCIDATING THE ROLE OF THE LEUCINE-RICH REPEAT KINASE 2 G2019S MUTATION IN CROHN’S DISEASE PATHOGENESIS USING A CITROBACTER RODENTIUM INFECTIOUS COLITIS MODEL

**DOI:** 10.1093/jcag/gwac036.178

**Published:** 2023-03-07

**Authors:** B Samman, J Yadav, G Bayer, E G Foerster, L Chen, J D B Rocha, S E Girardin, D J Philpott

**Affiliations:** 1 Laboratory Medicine and Pathobiology; 2 Immunology, University of Toronto; 3 Laboratory Medicine and Molecular Diagnostics, Sunnybrook Health Sciences Centre, Toronto, Canada

## Abstract

**Background:**

Associations have been found linking certain *LRRK2* kinase domain gain-of-function variants, such as G2019S, to the development of Crohn’s disease and Parkinson’s disease, yet their exact roles in pathogenesis remains elusive. LRRK2 is most robustly expressed in circulating and tissue-resident immune cells, such as neutrophils, lymphocytes, and macrophages. Myeloid cells deficient in LRRK2 exhibit defective antimicrobial responses, such as reduced production of reactive oxygen species in response to microbial stimuli and reduced bactericidal activity in response to infection. As an enteric colitis-inducing extracellular pathogen, *Citrobacter rodentium* can help us better understand the consequences of LRRK2 kinase hyperactivity on intestinal inflammation by correlating pathogen burden with key host response parameters over the course of infection.

**Purpose:**

To investigate the effects of the Crohn’s and Parkinson’s disease-associated Lrrk2 G2019S hyper-kinase mutation on pathogen burden and colonic inflammation in the context of *C. rodentium*-induced infectious colitis.

**Method:**

Wild-type and Lrrk2 G2019S mutant mice (7-8 weeks old) were fasted for 4 hours then infected with 1 x 10^8^ CFU of *C. rodentium* in a 3% NaHCO_3_ solution by oral gavage. Body weight, faecal pathogen burden, and faecal lipocalin-2 (Lcn2) concentrations were measured at 2, 4, 7, 9, 10, 12, and 14 days post-infection (DPI). Systemic pathogen burden (as measured in the mesenteric lymph nodes and spleen), colon length, colonic inflammatory gene expression, and histopathological scoring were assessed at 7, 10, and 14 DPI.

**Result(s):**

While G2019S mice exhibited marginally higher *C. rodentium* loads at certain timepoints, no significant differences were found in overall pathogen burden or pathogen clearance rates between genotypes over the first 14 days of infection. Faecal pathogen load peaked at 7-9 DPI in both WT and G2019S mice, which correlated with detectable levels of *C. rodentium* in the mesenteric lymph nodes and spleens of some mice at 7 DPI. Lcn2 secretion and the expression of inflammatory and antimicrobial genes of interest were induced robustly over the course of infection. They peaked and ebbed at timepoints correlating well with pathogen burden; however, no significant differences were observed between WT and G2019S mutant mice at the various timepoints assessed.

**Conclusion(s):**

Mice expressing the G2019S *Lrrk2* mutation exhibit neither defective pathogen control nor deleterious hyperinflammation – compared to WT mice – when infected with *C. rodentium*. Further research will aim to investigate the role of this *Lrrk2* variant in additional models of intestinal inflammation.

**Please acknowledge all funding agencies by checking the applicable boxes below:**

CCC, CIHR

**Disclosure of Interest:**

None Declared

